# Extending the sRNAome of Apple by Next-Generation Sequencing

**DOI:** 10.1371/journal.pone.0095782

**Published:** 2014-04-21

**Authors:** Marike Visser, Anelda P. van der Walt, Hans J. Maree, D. Jasper G. Rees, Johan T. Burger

**Affiliations:** 1 Biotechnology Platform, Agricultural Research Council, Pretoria, Gauteng, South Africa; 2 Department of Genetics, Stellenbosch University, Stellenbosch, Western Cape, South Africa; 3 Central Analytical Facilities, Stellenbosch University, Stellenbosch, Western Cape, South Africa; 4 Infruitec-Nietvoorbij, Agricultural Research Council, Stellenbosch, Western Cape, South Africa; NIGMS, NIH, United States of America

## Abstract

The global importance of apple as a fruit crop necessitates investigations into molecular aspects of the processes that influence fruit quality and yield, including plant development, fruit ripening and disease resistance. In order to study and understand biological processes it is essential to recognise the range of molecules, which influence these processes. Small non-coding RNAs are regulatory agents involved in diverse plant activities, ranging from development to stress response. The occurrence of these molecules in apple leaves was studied by means of next-generation sequencing. 85 novel microRNA (miRNA) gene loci were predicted and characterized along with known miRNA loci. Both cis- and trans-natural antisense transcript pairs were identified. Although the trans-overlapping regions were enriched in small RNA (sRNA) production, cis-overlaps did not seem to agree. More than 150 phased regions were also identified, and for a small subset of these, potential miRNAs that could initiate phasing, were revealed. Repeat-associated siRNAs, which are generated from repetitive genomic regions such as transposons, were also analysed. For this group almost all available repeat sequences, associated with the apple genome and present in Repbase, were found to produce siRNAs. Results from this study extend our current knowledge on apple sRNAs and their precursors significantly. A rich molecular resource has been created and is available to the research community to serve as a baseline for future studies.

## Introduction

Apple (*Malus* x *domestica*) is one of the world’s most important fruit crops. Due to the efforts of several individuals and working groups, a number of genomic resources have become available to form the basis for studying various biological processes in apple. These include the draft genome sequence (∼742.3 Mb), genome annotation, and various transcriptome datasets available in public databases, including datasets describing small RNAs, degradome, and expressed transcripts [1]–[6].

Plants acquired a variety of systems to regulate gene expression, including transcriptional (TGS) and post-transcriptional gene silencing (PTGS) [7], [8]. These regulatory processes can be triggered by double-stranded RNA (dsRNA) precursors that lead to the generation of small RNA (sRNA) molecules (∼17–26 nt), which target specific RNA molecules. One of the sRNA strands, known as the “guide strand”, associates with enzymes called Argonautes (AGOs) [9], as well as other proteins in either the RNA-induced silencing complex (RISC) [10] or the RNA-induced initiation of transcriptional gene silencing (RITS) complex [11]. Base-pairing to target nucleic acids complementary to the sRNA subsequently triggers silencing. Although silencing can result from DNA methylation and histone modifications, in plants it is most often the result of cleaving and degradation of the target RNA [12], [13].

The two dominant types of sRNAs in plants are microRNAs (miRNAs) and small interfering RNAs (siRNAs). miRNAs, despite not being the most abundant sRNA type, are the best-studied group. Primary miRNAs (pri-miRNA) are transcribed by RNA polymerase II from endogenous genes [14], [15]. These transcripts have a 5′-cap and a 3′-polyadenylated tail and fold into hairpin structures. Two successive cleavage reactions by Dicer-like (DCL) type III RNases result in the mature miRNA – a short (∼21 bp) double-stranded molecule containing a small number of mismatches between the miRNA and its antisense strand (previously known as miRNA*) [16], [17].

Small interfering RNAs are processed by DCL enzymes from long dsRNAs that are perfectly base-paired. This group can be divided into several sub-groups. See the review article by Axtell, for a comprehensive discussion on plant siRNAs [18]. The various siRNA species are produced via diverse biosynthetic pathways and affect gene regulation through different modes of action. One siRNA species, known as natural-antisense transcript siRNA (nat-siRNA), originate from the overlapping regions of complementary transcripts, which form dsRNA duplexes [19]. In contrast, the dsRNA precursors for phased-siRNA (phasiRNA) are generated by RNA-dependent RNA polymerase (RdRp) activity [20]. This group includes the well-characterized trans-acting siRNA (tasiRNA) [21]. The siRNA is spawned in a phased manner starting from the cleaved site. Repetitive genetic elements such as transposons and satellite DNA can also give rise to a group known as repeat-associated siRNAs (rasiRNAs) [22], [23]. Other more recently identified and less characterised functional sRNAs include species derived from small nucleolar RNA (snoRNA), ribosomal RNA (rRNA) and transfer RNA (tRNA) [24]–[28].

The role of sRNAs in the regulation of important biological processes is well documented. The present study improves the current apple sRNA species database, by adding and categorising novel and known sRNAs. A next-generation sequencing (NGS) approach was followed to sequence the sRNA transcriptome (sRNAome) of apple leaves. Computational analysis of the sRNA data provides a comprehensive resource to support future studies in order to investigate the role of specific miRNAs, phasiRNAs, nat-siRNAs, as well as rasiRNAs in various biological processes of apple.

## Results and Discussion

### Apple sRNA Next-generation Sequencing Data

**Figure 1:**
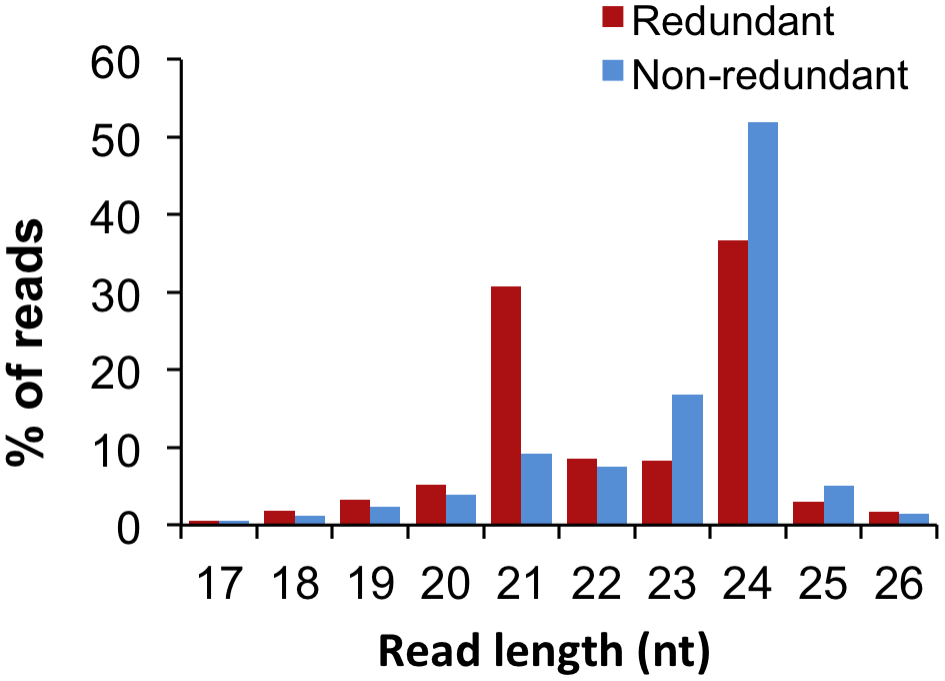
Sequencing library size distribution. Number of reads, 17 to 26****nt in length, as a percentage of either the total redundant or non-redundant reads in this size range.

The ability of NGS to detect low titres of sRNA species in plant cells was exploited to determine the sRNAome of apple leaves. Of the 71,273,331 high quality sequence reads, 96.58% were 17 to 26 nt in length (Table 1). The majority of functional sRNA species involved in TGS and PTGS is considered to fall within this range. The library was dominated by reads 24 nt in length (37%) followed by 21 nt long reads (31%), which also displayed the greatest redundancy (95%) (Figure 1). This high level of redundancy may be attributed to a small group of 21 nt long sRNAs with an elevated demand by the cell. sRNAs that often fall into this size group are miRNAs and phasiRNAs. Analysis of the miRNAs (Table 1) showed a high level of redundancy, although the miRNA group alone cannot fully explain the redundancy of the 21-nt size group. The dominance of the two size groups highlighted their probable significance in regulating biological processes. sRNAs from these size groups include heterochromatic siRNAs, which are 24 nt in length and function by means of RNA-mediated methylation of DNA targets [13], as well as phasiRNAs and miRNAs, which can be of either length. It is important to note that miRNAs are not necessarily restricted to a length of 21 or 24 nt, but have been found to range between 20 and 24 nt in length.

**Table 1:** Summary of the sequenced reads.

Small RNA	Unique	Total
Adapter trimmed	14,027,369	77,651,426
High quality reads	12,969,231	71,273,331
17–26 nt reads	12,422,959	68,837,477
miRNA reads^a^	249	12,119,076
natsiRNA reads	108,657	813,241
phasiRNA reads^b^	363	30,500
rasiRNA reads	1,139,528	5,526,689

a Reads with perfect matches to known and novel mdm-miRNAs.

b Reads with perfect matches to phasiRNA which are in phase with miRNA cleave-sites.

### Detection of Known miRNAs

To identify known miRNAs present in the dataset, the reads were compared to the publicly available miRNA Registry Database, miRBase (version 20) [3]. When only allowing perfect matches, 11,847,841 reads mapped to apple miRNAs (mdm-miRNAs) recorded in miRBase. The database contains 207 mature mdm-miRNAs (from 43 families), which were predicted from Golden Delicious plants. During this study, 195 of the listed mdm-miRNAs, belonging to 40 families, could be detected in leaf material (Table S1A). No members were detected for the miR828, miR2111 and miR7128 families. The abundance of individual known miRNAs ranged from single reads to a few million, with most (75.9%) of the mature mdm-miRNAs having a read count of greater than 100. The miRNA cluster with the highest read count, mdm-miR166a-i, accounted for 91.51% of all the reads mapped to known apple miRNAs. In total, miR166 was the largest represented miRNA family followed by miR396 and miR398 (Table S1B). This result differs from a study by Xia et al. [5], who found miR167 to be the most abundant in leaf sequencing data, closely followed by miR165/166. However, differences in expression levels of miRNA families between studies can be expected and may be attributed to differences in developmental stages, on-going physiological processes, and environmental conditions. mdm-miR166 was previously proven to target apple homeobox-leucine zipper proteins [5]. This protein family is involved in a range of plant processes including growth and morphogenesis [29]. The vast number of miR166 reads, mirroring their high expression level, is a clear indication of the central role of this miRNA species in regulating apple processes.

Besides known mdm-miRNAs, 77 unique reads with 100% homology to miRNAs from other plant species, not yet identified in apple, were also detected (Table S1C). These reads numbered 198,840, with the highest represented sequence having an individual read count of 180,963. Twenty of the reads homologous to non-apple Viridiplantae species had a sum of more than 100. However, the presence of homologous sequences in the apple sRNA dataset is not sufficient evidence for these to be considered apple miRNAs. This matter can possibly be resolved by analysing their region of origin on the apple genome during novel miRNA prediction.

### Novel miRNA and Target Prediction

**Figure 2:**
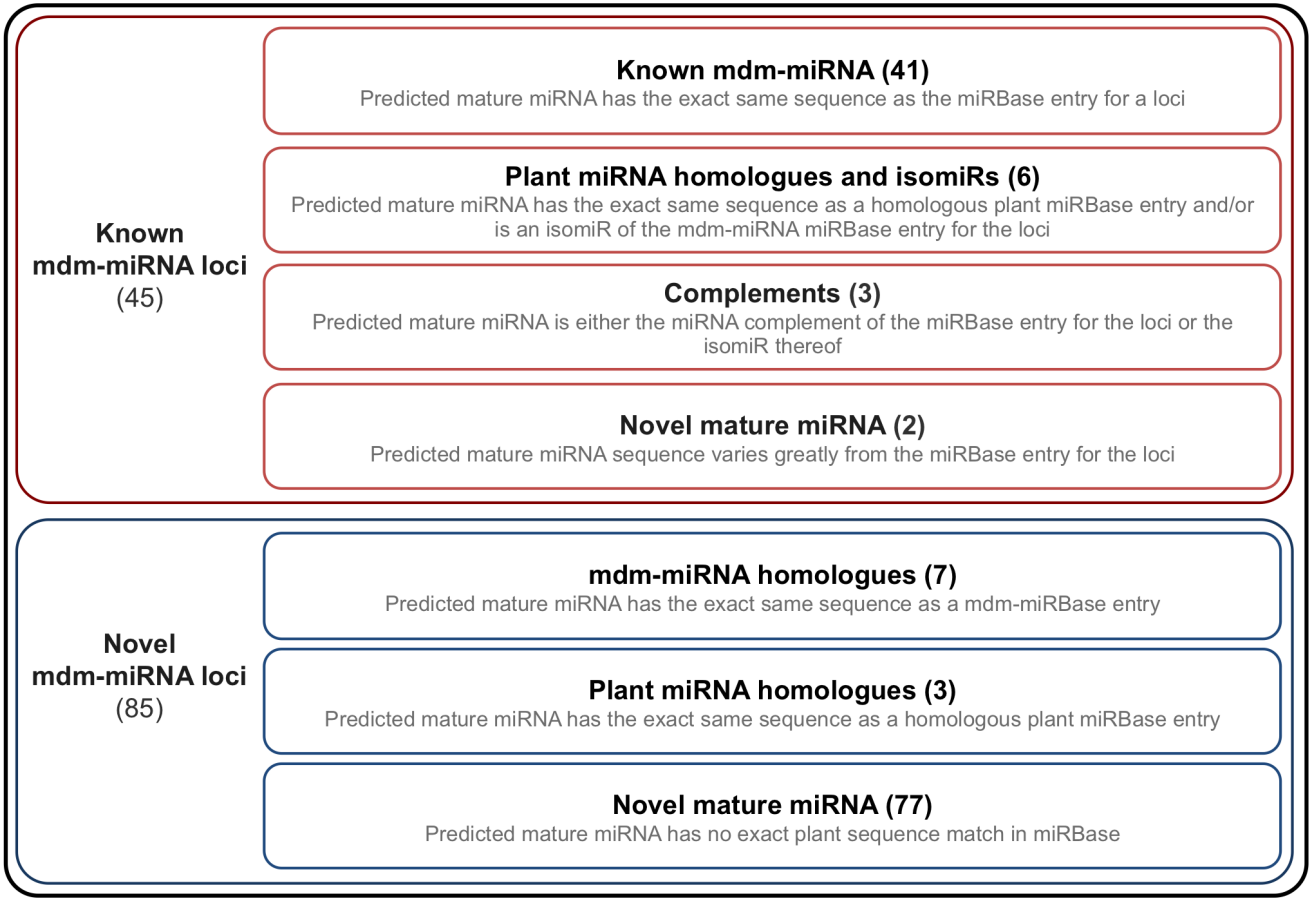
Known and novel miRNA predictions. Diagram defining the different classifications used for known and novel mdm-miRNA loci predictions. The predicted mature miRNA at known apple miRNA loci belonged to one of four classes: it could have the same sequence as the mature apple miRBase entry; it could have the same sequence as another plant homologue which can also be an isomiR of the apple miRBase entry; it could be the antisense-complement of the miRBase entry or an isomiR thereof; or it could have a sequence that varies significantly from the miRBase entry and therefore is classified as a novel miRNA. Novel miRNA loci had mature miRNAs, which belonged to one of three classes: it could have a sequence which is the same as another apple miRNA already present in miRBase and may therefore fall into the same family, it could have a sequence which is the same as a homologous plant miRBase entry; or it could have a sequence for which there is no exact plant sequence entry in miRBase.

**Figure 3:**
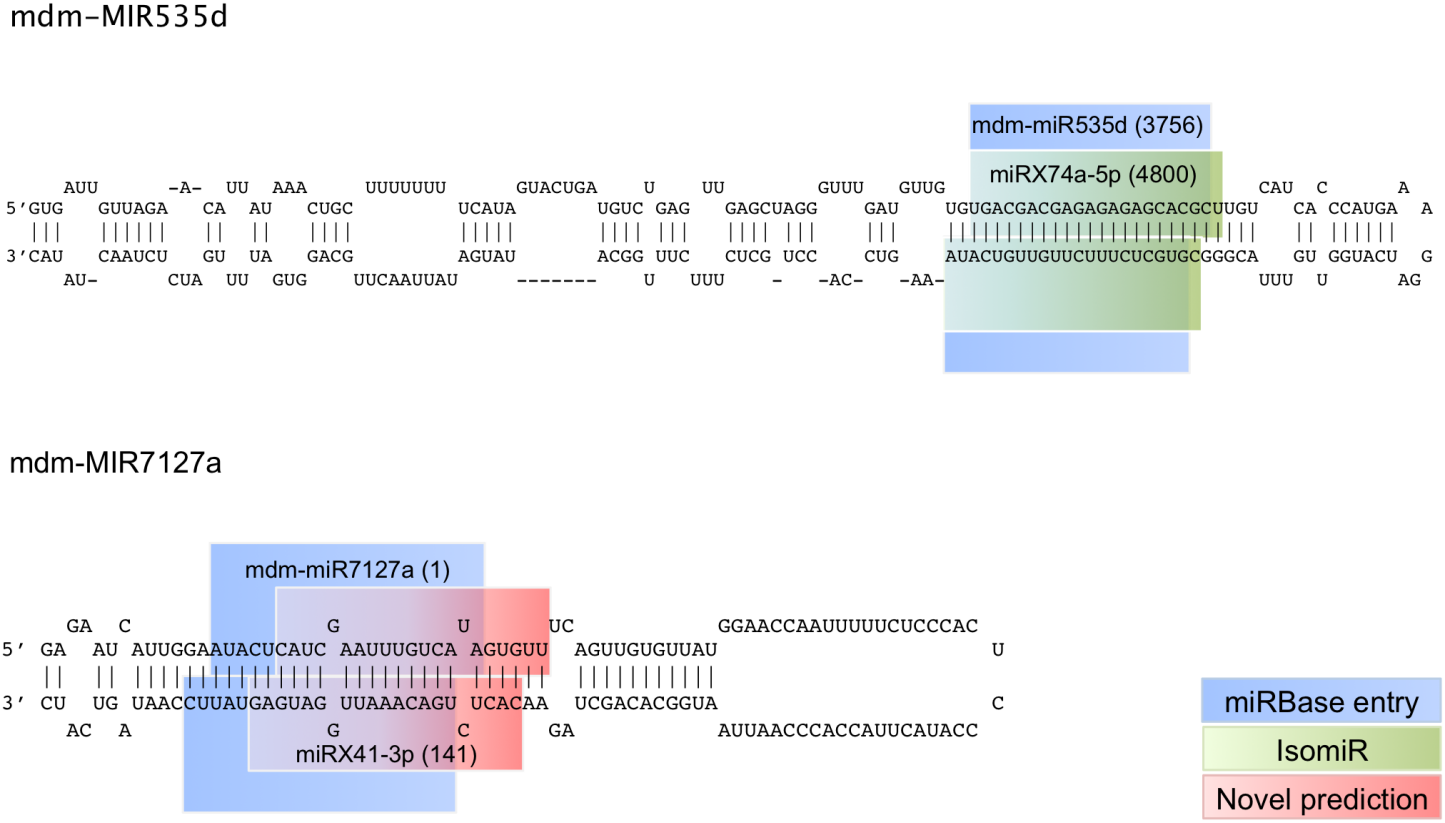
miRNA examples. (A) Example of a miRNA precursor for which the miRBase mature entry, as well as its isomiR was predicted as mature miRNAs. (B) Example of a miRNA precursor for which the miRBase mature entry was not predicted as a mature miRNA, but rather a mature miRNA varying significantly from the miRBase entry and was therefore classified as a novel miRNA. Read counts are given in brackets.

Due to the essential regulatory role that miRNAs play in many biological processes it is important to expand the available miRNA knowledge base. To identify novel apple miRNAs we performed a miRBase-independent, computational miRNA prediction analysis, based on the sRNA sequencing data. 130 genomic loci were predicted to be miRNA genes, each having a mature miRNA represented by at least 10 reads (Figure 2 and Table S1D). Nine of these miRNA genes have more than one potential mature miRNA pair. The predicted genomic regions of 45 of the miRNA precursors overlapped with the loci of known mdm-miRNAs. For the majority of these precursors, at least one of the predicted mature miRNAs was a known mdm-miRNA. For two of the precursors, mdm-MIR399e and mdm-MIR5225c, the current analysis predicted the complement of the mature miRBase entry sequence as the novel miRNA. Some of the predicted novel mature miRNAs were isomiRs (sequence variants) of existing miRBase mature entries, some of which were homologous to miRNAs from other plant species. At four of these known miRNA gene loci, the newly predicted miRNAs had read counts that were higher than those of the current miRBase entry. Three of these miRNAs were homologous to miRNAs from other plant species. Figure 3 illustrates two cases where the mature sequence differed from the miRBase entry or where the isomiR was predicted as mature along with the miRBase entry. The fact that the mature sequence, as registered in miRBase, does not correspond to the dominant miRNA for the precursor in this dataset does not necessarily imply that the registry entry is a miss-annotation. As can be seen from the data by Xia et al. [5], different isomiRs can be expressed at different levels relative to each other depending on the tissue type. Similar variation in expression levels can probably also be ascribed to differences in environmental conditions.

In addition to the known mdm-miRNA gene loci, 85 putatively novel precursor miRNA loci were identified. The mature miRNAs predicted for a few of these novel loci were the same as known mdm-miRNAs and can therefor be considered new members of the already known mdm-miRNA gene family. Along with novel miRNA loci also having a novel mature sequence, additional precursors were identified with predicted mature sequences homologous to miRNAs from other plant species.

Of the predicted miRNA loci, 33 overlapped with predicted apple transcripts. Although plant miRNAs predominantly originate from intergenic regions, it was demonstrated earlier that they can also be derived from gene introns, known as mirtrons [30], and exons [31].

*In silico* analysis with psRNATarget predicted targets for 217 of the novel miRNAs. 81.9% of all targets were predicted to be down-regulated through cleavage (Table S1E). Additional analysis with TargetFinder and CleaveLand, applying a Golden Delicious degradome sequencing dataset, resulted in the successful validation of 26 cleaved mRNA targets (Table S1F). A dataset generated from the cleaved RNAs of different Golden Delicious tissue types was used to validate miRNA targets (accession no. SRR413929) [5]. Despite the fact that the publicly available degradome dataset was generated from the same apple variety as the miRNA dataset in the present study, differences in environmental conditions may have prevented the validation of a larger number of computationally-predicted miRNA targets. Another limitation of the degradome dataset is the fact that it was generated from a range of different plant tissues, which may have caused the under-representation of leaf material in the sample.

### NAT and nat-siRNA Identification

**Figure 4:**
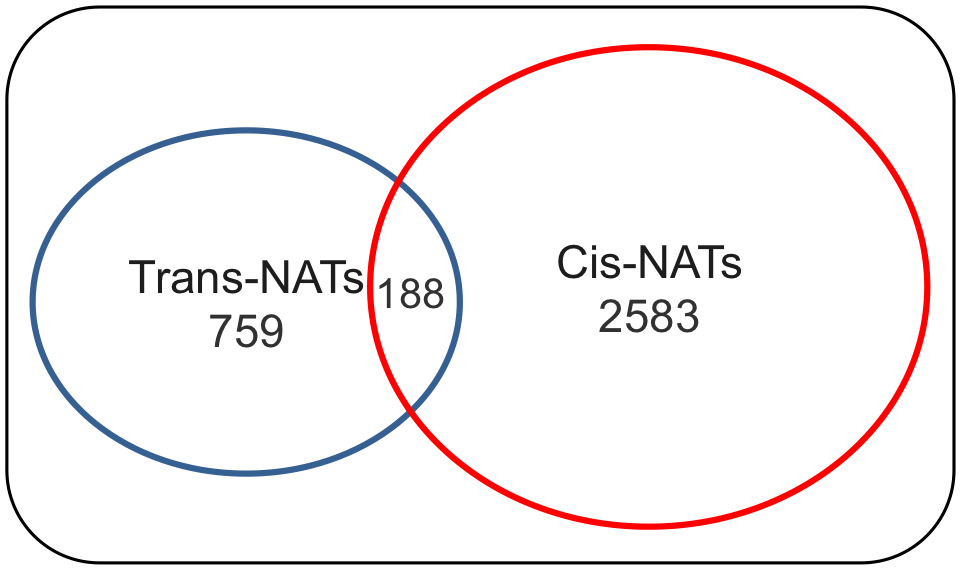
Transcripts forming cis- and trans-NAT pairs. Diagram illustrating the number of apple transcripts involved in either a cis- or trans-NAT relationship as well as the number of transcripts which are shared by the two groups of NATs.

Earlier studies have expounded the contribution of natural antisense transcript (NAT) siRNAs (nat-siRNAs) in plant development [32], [33], disease resistance [34], [35] and stress responses [19]. nat-siRNAs are processed from the overlapping region of transcript hybrids and in general down-regulate the expression of one of the transcripts involved in the duplex [36]. We have identified 1423 cis-NAT and 2198 trans-NAT pairs, of which 19 and 3 pairs, respectively, contained more than 1 overlapping region (Table S2A and S2B). The Genome Database for Rosaceae (GDR) [1], [2] contains a total of 63541 predicted apple transcripts, of which 3752 were predicted to be part of various combinations of NAT pairs. Of all transcripts, 4.4% were involved in cis-NATs and 1.5% in trans-NATs. A small subset of transcripts (5% of all NATs) could form both kinds of NAT pairs (Figure 4), similar to what was found in other studies [37]–[39].

**Figure 5:**
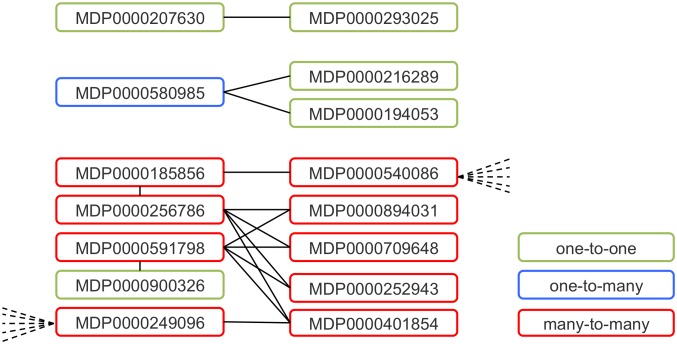
Natural antisense transcript networks. Diagram illustrating the three different relationships a NAT can be involved in i.e. a one-to-one (green), one-to-many (blue) or many-to-may (red) relationship. Solid lines indicate NAT pairs while dashed lines indicate a NAT relationship with a transcript not shown in the diagram.

A single transcript can be part of a duplex in a one-to-one (i.e. can form a duplex with only one other transcript), one-to-many or many-to-many relationship [37]–[40]. Figure 5 illustrates these criteria with reference to results from the present study. In our analysis, 81.5% of the NATs were involved in one-to-one, 4.6% in one-to-many and 13.9% in many-to-many bonds. These figures were 41.6%, 13.7% and 44.7% for trans-NATs, and 90.7%, 5.2% and 4.1% for cis-NATs, respectively. This indicates that NATs are part of a complex gene regulatory network in apple, similar to what has been observed in other plants [38]. Although these computationally-predicted NATs have the potential to hybridize *in planta,* concurrent expression in the same cellular location must occur for these duplexes to form.

**Figure 6:**
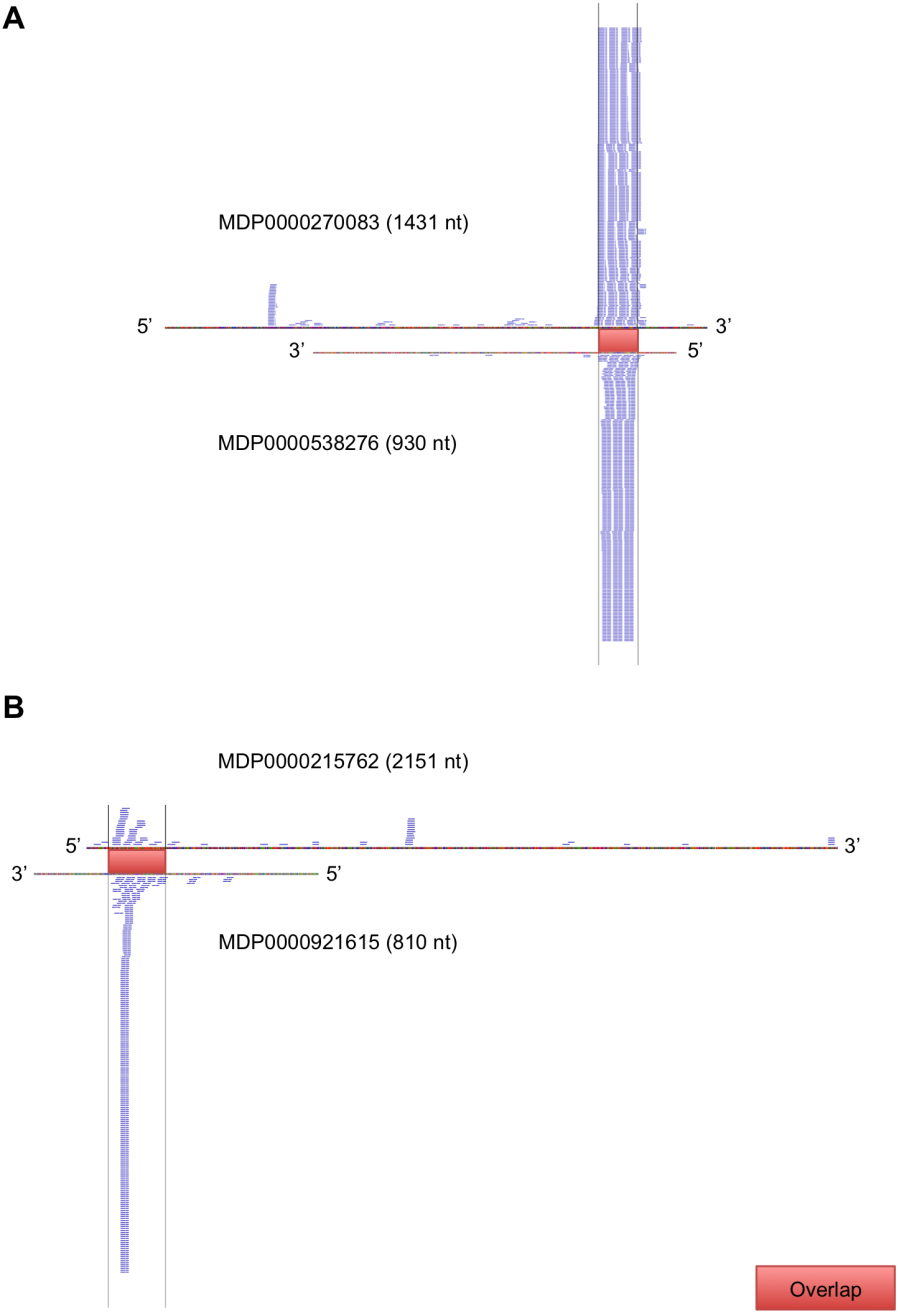
siRNA enrichment of trans-NAT overlaps. Illustration showing two trans-NAT pairs predominantly producing siRNAs from their overlapping regions.

In order to determine whether the siRNA spawned from overlapping regions was not purely by chance, the sRNA density (number of reads per kb of transcript) of the overlaps was compared to that of the rest of the NATs. The median of the siRNA densities of the overlapping regions of cis-NATs was 6.7 reads/kb while the reads on the overlapping regions of the trans-NATs had a density of 299,700 reads/kb (Table 2). Previous studies demonstrated that cis-NATs of protein-coding genes generally yield low levels of sRNAs when compared to non-cis-NATs [41], [42]. Mapping analysis indicated that trans-NATs of apple are significantly enriched for sRNAs in their overlap regions when compared to the rest of the transcripts (p<2.2×10^−16^). Two examples of this enrichment are given in Figure 6. Conversely, the cis-NATs did not produce more sRNAs from their overlaps, it rather showed a reduction in sRNA generation (p<2.2×10^−16^). This is not in accordance with what has been found for other plant species by Zhou et al. and Henz et al. [38], [41]. It is important to note that while these studies found an increase in sRNA production from the cis-NAT overlapping regions when compared to the non-overlapping regions of the NATs, they found no significant difference between siRNA production from cis-NATs and transcripts uninvolved in NAT formation. The latter study could not find any evidence to support the regulation of cis-NAT by siRNA more than was the case for any non-overlapping transcript.

**Table 2:** Natural-antisense transcript summary.

	Cis-NAT	Trans-NAT	Unclassified
**Pairs**	1423	2198	812
**Portion of total transcripts (%)**	4.4	1.5	0.35
**Overlap length (nt)** a	367	124	116
**One-to-one (%)**	90.7	41.6	68.3
**One-to-many (%)**	5.2	13.7	2.7
**Many-to-many (%)**	4.1	44.7	29
**Density** b **in overlap/transcripts**	6.7/23.7	299,700/1,172	305,300/1,600
**Overlap enrichment (p-value)**	No (<2.2×10^−16^)	Yes (<2.2×10^−16^)	Yes (<2.2×10^−16^)
**>2-fold stand bias (%)**	75.5	42.7	47.1

a Values indicate the median.

b Reads/kb.

To allow for the down regulation of a transcript, the expression of the complementary transcript is first increased to stimulate nat-siRNA formation, which will target the constitutively expressed transcript [19], [34], [36], [43]. This anti-correlation of transcripts [33], [35], [41] is not always observed [41]. Due to the absence of quantitative transcriptome data in the current study, it was not possible to analyse correlation of expression for paired transcripts. However, we did observe a strand-bias in the nat-siRNAs, of at least two-fold, for more than 53% of the NATs. Our data indicate that, for a significant number NAT pairs, the siRNAs were derived predominantly from one of the NATs, and thus suggests the preferred down-regulation thereof. These results support those of similar studies [35], [38], [39].

Although an earlier study has reported on apple trans-NATs [44], to our knowledge, this study is the first to report on apple cis-NATs and also the first to use annotated transcripts to investigate the production of NATs and nat-siRNAs in apple. The overlapping regions and nat-siRNAs identified here can be combined with transcriptome data in future studies to investigate gene regulation in which transcript hybridisation plays a central role. Besides cis- and trans-NATs a number of NAT pairs were also identified for which the chromosomal coordinates of at least one of the transcripts are unknown (Table S2C). These transcript pairs were therefor grouped into a third unclassified group, which followed the same trend as the trans-NAT group (Table 2).

### PHAS Identification and phasiRNA Analysis

sRNAs produced from trans-acting siRNA genes (*TAS*), were first considered to only work in trans (hence the name tasiRNA). Subsequently, their cis-action was also suggested [45], [46]. For this reason Zhai et al. introduce the term phasiRNA for all phased siRNAs, irrespective of whether they target other transcripts in trans or their own source [20]. The genes were called *PHAS* genes and included protein-coding as well as non-coding genes.

Trans-acting siRNA genes are involved in plant development [47], [48], biotic stress [35] and abiotic stress [28], [36], [49]. Several *TAS* gene families have been recognized in diverse plant species, some conserved and some species-specific [45], [50], [51]. A recent study by Xia et al. have identified and characterised the *TAS3* and *TAS4* families in apple [5]. They also discovered myeloblastosis (*MYB*) genes from which phasiRNAs were generated after cleavage by miR828. The same research group later characterised an additional novel *TAS* gene in apple, which they called Md-*TASL1* [52].

In the current study two approaches were followed to identify phased regions (clusters) in apple, by firstly implementing transcript data and secondly the genome. In total 157, 21 nt phased clusters were predicted to be statistically significant (Table S3A and S3B). Four of the transcripts were reported before to produce phasiRNAs, namely MDP0000578193 and MDP0000124555 [5], as well as MDP0000179176 and MDP0000302095 [52]. When the phased regions were examined using the NCBI BLAST database the majority of these aligned against disease-responsive genes, particularly genes belonging to protein families with nucleotide binding site leucine-rich repeats (NB-LRR) domains. The production of phasiRNAs was demonstrated earlier for NB-LRR protein families [20], [50], [53]–[55], as well as for pentatricopeptide repeat (PPR) [50], [52], [53], [56], MYB [5], [57], [58] and Auxin Signalling F-Box (AFB) protein families [5], [50]. All of these protein families also displayed phasiRNA generation in this study. Furthermore, besides being a source of phasiRNAs, pathogen resistance genes are also known to be targeted by this sRNA species [35], [50], [53], [56], [59], indicating the importance of phasiRNAs in integrated networks for gene regulation. BLAST hits included not only the above-mentioned protein-coding genes, but also *TAS3* gene homologues as expected.

When comparing the results for the two phasing analysis approaches, the genomic coordinates for a large number of phased transcript regions overlapped with that of phased genomic regions. This can be expected for phased regions, which do not span an intron. Not all transcripts are anchored onto the genome assembly used, which can additionally cause phased transcript regions to appear absent from the genomic results.

miRNAs can act as phase-initiators. After target cleavage dsRNA is formed through RdRp activity followed by phasiRNA generation from the cleaved site [45]. To identify potential phase-initiators, miRNA targeting phased clusters were identified. Despite a number of miRNAs potentially targeting the phased clusters, only 26 had a miRNA target cleavage site that fell into the dominant phasing register (Table S3C and S3D). Previous studies have also shown phased regions with a phase-initiating cleave site being out-of-phase [5], [50], [52], [57], [60], [61]. These instances, known as phase-drift [50], [62], can be ascribed to DCL slippage leading to a slight shift in the phasing with regards to the cleaved site [50], or to the presence of an additional cleavage of the phased region by a phasiRNA [60], [61]. In this study, the cleavage start sites for 10 clusters were validated with the apple degradome dataset (Table S3C and S3D). As mentioned previously, the differences in experimental conditions between the degradome sequencing and the sRNA-sequencing in this study may explain the lack of miRNA cleavage validation. Almost all (24 out of 29) initiator-miRNAs were 22 nt in length and had a uracil at the 5′ end. The length of the sRNA initiator play a role in the phasing model. The phasing model can be based on either single (one-hit) or double (two-hit) miRNA target sites [60]. Although 22 nt miRNAs are mostly considered to be involved in phasing triggered by a single miRNA site [63], their association in the two-hit phase model was also demonstrated [20], [52]. All the 22 nt miRNAs in this study complied to the one-hit phase model.

### rasiRNA Identification

This study demonstrated that 517 of the 524 repetitive sequence entries (Repbase *M*. *x domestica*) spawn sRNAs (Table S4). These entries included satellite DNA and integrated virus sequences, as well as retro- and DNA transposable element (TE) sequences. The largest cluster of reads (550,108 reads) mapped to retrotransposon-1 (RTE-1) followed by RTE-1B and DNA-transposon9–10. Generally, no particular strand-bias (more than 2-fold difference) was observed when investigating rasiRNAs mapping to the repeats. Some siRNA clusters, e.g. the long terminal repeat Copia-23, had a strong bias towards one of the repeat strands (more than 200-fold difference). The bulk (>50%) of rasiRNAs were 24 nt in length, a size-group frequently linked to heterochromatin-associated siRNAs [64]. Satellite 1 DNA is known to be associated with heterochromatin in the centromeres and other chromosomal regions [65]. Therefore it can be suggested that the siRNAs, which mapped to SAT1, can be classified as heterochromatic siRNAs. This group formed the sixth largest cluster of rasiRNAs (180,856). In our analysis, 9.2% and 8.5% of the rasiRNAs were 21 and 22 nt in length respectively and have also been implicated in TE silencing before [53], [66], [67].

## Conclusion

The roles of small RNAs in gene regulation, and therefore in biological processes are being investigated for most important agricultural crops, including woody fruit crops. This study provides the most comprehensive single report on the sRNAs of apple. The apple miRNA database was extended significantly through the prediction of 85 novel miRNA precursor loci. Characterisation of these novel, and known loci, revealed mature miRNAs that were either known (mdm-miRNAs, miRBase 20), known plant homologues, or novel. Cis- and trans-NAT pairs, and the associated nat-siRNAs produced from their overlapping regions, were identified. Phased regions were identified at both the genome and transcriptome level. Besides non-coding loci, a number of protein-coding genes were shown to produce phasiRNAs. Finally, rasiRNAs for nearly all the apple repeat sequences in Repbase were identified.

This study, through NGS and computational analysis, identified a range of novel and known sRNA species in apple. Collectively they significantly add to the existing databases and will provide a platform for future functional studies in this important fruit crop.

## Methods

### NGS and sRNA Dataset Preparation

Sample material was collected from six, greenhouse grown, *M*. *x domestica* cv. Golden Delicious (NIVV) seedlings, grafted onto MM.109 rootstocks. Total RNA was extracted from leaf material using the Plant RNA Reagent Kit (Invitrogen) and the small RNA fraction (17–29 nt) was purified from total RNA using a 15% TBE-urea polyacrylamide gel. Library preparation was performed by means of the TruSeq Small RNA library preparation kit from Illumina, and sequenced on an Illumina HiScan SQ instrument. The sequence data from the six libraries were pooled. The software cutadapt (V 1.0) [68] was applied to remove adapter sequences and the reads were filtered for quality (phred score ≥20) using FASTX-toolkit (V 0.0.13, http://hannonlab.cshl.edu/fastx_toolkit/index.html). Only reads 17–26 nt in length were used for sRNA analysis. The adapter-trimmed libraries (unfiltered), of the separate six samples, were submitted to the NCBI-SRA database (accession no. SRR1136652 to SRR1136657).

### miRNA Analysis and Target Prediction

Known apple miRNAs as well as sRNAs homologous to known miRNAs of other species were identified using miRanalyzer (V 03/2012) [69], [70]. To get an indication of the sRNA reads which represent an exact miRNA in the registry, no mismatches were allowed to miRBase entries, thus excluding any isomiRs. The “Plant mode” of ShortStack (V 0.4.1) [71] was used to perform novel miRNA prediction from sRNAs that were read-mapped, with a maximum of one mismatch to the *M. x domestica* genome primary pseudo-haplotype assembly (*M*. x *domestica* Whole Genome v1.0p) [1], [2]. ShortStack filters predicted hairpin structures to identify miRNA precursors following the plant miRNA criteria as set by Meyers et al. [72]. It allows a maximum of 150 base pairs in a miRNA hairpin, a maximum of five unpaired nt in a mature miRNA duplex, unlimited loop length and a minimum fraction of 0.8 mappings within Dicer size range to annotate a locus as Dicer-derived.

Targets for the newly predicted miRNAs were first accessed using the web-based tool psRNATarget (http://plantgrn.noble.org/psRNATarget/) [73], applying default parameters. In an attempt to identify mRNA cleaved by the novel miRNA TargetFinder (V 1.6, http://carringtonlab.org/resources/targetfinder) along with CleaveLand (V 3.0.1) [74] was utilized to predict and validate miRNA cleavage sites. The apple degradome library used for validation was obtained from the NCBI-SRA database (accession no. SRR413929).

### Nat-siRNA Identification

Cis- and trans-natural antisense transcripts were identified following a similar workflow to Zhou et al. [38]. Apple transcript sequences (*M*. x *domestica* v1.0 consensus CDS 300 flanking) were obtained from the GDR (http://www.rosaceae.org/species/malus/malus_x_domestica) [1], [2]. Transcript sequences included coding regions as well as up to 300 nt up- and downstream. Duplex formation of overlapping genomic regions (>50 nt) formed by transcripts originating from opposite strands was validated using UNAfold (V 3.8) [75]. These hybridizing molecules were considered to be cis-NAT pairs. Trans-natural antisense transcripts were identified by aligning the transcripts to each other using standalone BLAST (V 2.2.27+)[76]. The trans-NAT pairs were derived from diverse genomic regions, with an overlapping region of more than 100 nt having 100% identity. The same analysis, as for trans-NAT, was performed on transcripts for which the genomic region was unknown. UNAfold was again used to validate duplex formation.

The density of the sRNAs on the overlapping and non-overlapping regions of the NATs was compared to determine whether the overlapping regions of the NATs were significantly enriched with sRNAs. The density was determined by calculating the number of reads per kilobase of overlapping or non-overlapping NAT regions while the significance of the difference in densities was determined by mean of a Wilcoxon rank sum test.

### Phased Cluster and siRNA Identification

Phased regions were identified using ShortStack [71], allowing a single mismatch of the sRNA to either the apple genome or computationally-predicted transcriptome. P-values were corrected for multiple testing and a Bonferroni adjusted significance level of 0.0034 or 0.001 was used for transcript and genomic analysis, respectively. Potential phase-initiating miRNAs were identified using psRNATarget [73] and cleavage at the target site was validated with an apple degradome sequencing dataset from the NCBI-SRA database (accession no. SRR413929) as described by Zhang et al. [51].

### rasiRNA Identification

miRanalyzer was used to identify rasiRNAs based on *M*. *x domestica* repeat sequences present in Repbase 17.12 [77], [78]. After removing sequences that matched known mdm-miRNAs, a single mismatch was allowed between the sRNA read and the repeat sequence.

## Supporting Information

Table S1 miRNA results. The number of sRNA reads associated with apple miRBase entries and families, as well as other plant homologues. Predicted miRNA loci (with their properties) along with novel miRNA target prediction and degradome validation results are also given.
(XLSX)

Table S2 NAT results. Cis-, trans- and unclassified apple natural antisense transcript pairs with the sequence and coordinates of the overlapping regions.
(XLSX)

Table S3 phasiRNA results. Phased genomic and transcript regions with their properties such as phase-initiating miRNA, strandedness, phase-offset and alignment results for the region.
(XLSX)

Table S4 rasiRNAs results**.** The number of sRNA reads associated with both strands of apple repeat sequences in Repbase.
(XLSX)
